# Pelvic Floor Reconstruction After Radical Prostatectomy: A Systematic Review and Meta-analysis of Different Surgical Techniques

**DOI:** 10.1038/s41598-017-02991-8

**Published:** 2017-06-02

**Authors:** Jianfeng Cui, Hu Guo, Yan Li, Shouzhen Chen, Yaofeng Zhu, Shiyu Wang, Yong Wang, Xigao Liu, Wenbo Wang, Jie Han, Pengxiang Chen, Shuping Nie, Gang Yin, Benkang Shi

**Affiliations:** 1 Department of Urology, Qilu Hospital of Shandong University, Jinan, Shandong 250012 P.R. China; 2 Department of Endocrinology and Metabolism, Shandong Provincial Hospital affiliated to Shandong University, Jinan, Shandong 250021 P.R. China; 3 Department of Radiation Oncology, Shandong Cancer Hospital and Institute affiliated to Shandong University, Jinan, Shandong China; 4 Department of Radiation Oncology, Qilu Hospital of Shandong University, Jinan, Shandong 250012 P.R. China; 5 Clinical Epidemiology Unit, Qilu Hospital of Shandong University, Jinan, Shandong 250012 P.R. China

**Keywords:** Prostate cancer, Surgical oncology, Prostate

## Abstract

Radical prostatectomy (RP) is the gold standard for the treatment of localized PCa. A meta-analysis was conducted to evaluate the effect of different techniques of pelvic floor reconstruction on urinary continence. A comprehensive search was made for trials that evaluated the efficacy of pelvic floor reconstruction. Relevant databases included PubMed, Embase, Cochrane, Ovid, Web of Science databases and relevant trials from the references. Random-effects model was used to estimate risk ratios (RRs) statistics. Pooled results of patients treated with posterior reconstruction (PR) demonstrated complete urinary continence improved at 1–4, 28–42, 90, 180 and 360 days following catheter removal. Anterior suspension (AS) was associated with improvement only at 28–42 days. The anterior reconstruction (AR) + PR was associated with urinary continence at 1–4, 90 and 180 days. AS + PR was not associated with any benefit. And PR improved social urinary continence at 7–14 and 28–42 days. No benefit was associated with AS. AR + PR had better outcomes at 90 and 180 days. AS + PR was significant improved at 28–42 and 90 days. Patients who underwent RP and PR had the least urinary incontinence. No significant benefit was observed after AS. AR + PR and AS + PR had little benefit in the post-operative period.

## Introduction

Prostate cancer (PCa) is the most common cancer, with an incidence of approximately 21% in the general population. It is the second most common cause of male cancer death in the world, affecting about 8% of men^1^. By 2016 in the United States 180,890 new PCa cases and 26,120 deaths from PCa are predicted to occur^1^. Radical prostatectomy (RP) is the gold standard for the treatment of localized prostate cancer. Robot-assisted radical prostatectomy (RARP) and laparoscopic radical prostatectomy (LRP) are widely used, and have been associated with lower positive surgical margin rates, shorter hospitalizations, lower post-operative leakage rates, lower transfusion requirements and a shorter period of urinary catheterization^2^. Early urinary incontinence remains one of the most common complications after RP.

Post-operative urinary incontinence is severely bothersome^3^ and is associated with a decreased quality of life. Urinary incontinence is often perceived as more bothersome than erectile dysfunction^4^. Several methods of pelvic floor reconstruction have been introduced to reduce the risk of urinary incontinence. Posterior reconstruction (PR) of the rhabdosphincter was initially described by Walsh^5^ and later popularized by Rocoo *et al*.^6, 7^. It is still a popular technique for controlling urinary incontinence. Anterior reconstruction (AR) was introduced by Tewari *et al*.^8^ and later combined with PR to yield an incremental benefit (AR + PR)^9–11^. A simple anterior suspension (AS) technique using sutures anchored to the pubic bone was first described by Sugimura *et al*. to improve early urinary continence^12^. The effect of anterior suspension combined with posterior reconstruction (AS + PR) has also been examined.

Now the effect of different surgical techniques for improving urinary continence is not clear yet. Rocco *et al*.^13^ reported a meta-analysis of posterior reconstruction technique and several trials have been conducted to evaluate the time to urinary continence after LRP and RARP. However, the previous study didn’t evaluate other surgical techniques. The publication of new studies evaluating PR, AS, AR + PR, and AS + PR add to the power of a meta-analysis. We conducted a meta-analysis evaluating the continence rate at different time intervals after different surgical techniques.

## Results

**Figure 1:**
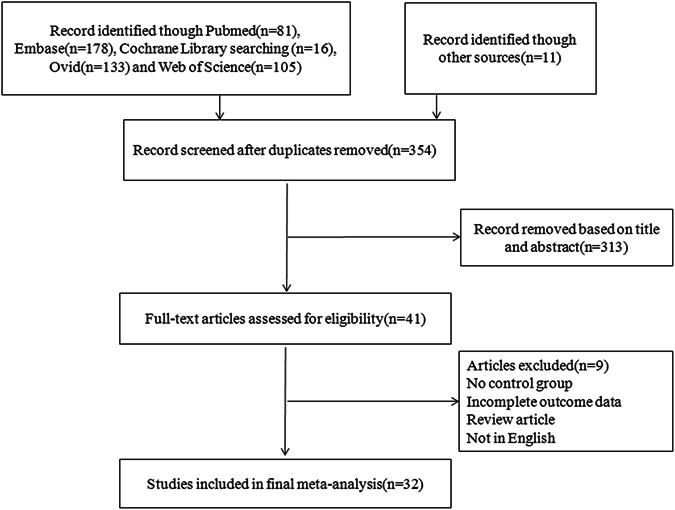
Selecting the flowchart for the inclusion of studies in the meta-analysis.

354 trials were identified by reviewing abstracts and articles. 159 duplicates were removed. Nine additional trials were excluded because there was no comparison group, outcome data was incomplete, it was a review article, or the article was not in English. The final set of trials eligible for analysis included 32 studies for the qualitative analysis^7, 9–12, 14–40^. The selection strategy is shown in Fig. 1. The characteristics of the included trials are outlined in Table 1. A total of 4697 patients were included in this meta-analysis. 19 trials^7, 15–32^ evaluated the efficacy of PR, 7 trials^12, 33–38^ evaluated the efficacy of AS, 4 trials^9–11, 14^ evaluated the efficacy of PR + AR, and 2 trials^39, 40^ evaluated the efficacy of PR + AS. Seven of these trials were RCTs^9, 15, 31, 32, 37, 38, 40^. Six trials^11, 18, 25, 29, 32, 33^ evaluated IPSS and EPIC urinary domain scores.

**Table 1:** Characteristics of the included studies.

Study (Year)	Country	Study period	Study design	Technique	Definition of continence	Evaluation of continence	Nerve sparing	No. Patient S/C	Main outcomes S/C
Francesco Rocco^6^	Italy	1998–2003	Historical Cohort Study	PR(RRP)	0 pad	ICIQ-SF	N/A	161/50	3 day: 72.0%/14.0% 1 mon: 78.8%/30.0% 3 mon: 86.3%/46.0%
U. Anceschi^15^	Italy	2007–2012	Historical Cohort Study	PR(LRP)	0 pad	ICQ-SF and SF-36	N/A	52/54	1 mo: 69%/37% 3 mons: 86%/54%
Rafael Coelho^16^	USA	N/A	Historical Cohort Study	PR(RALP)	0 pad	EPIC	+	473/330	1 wk: 28.7%/22.7% 4 wks: 51.6%/42.7%
Georgios Daouacher^17^	Sweden	2005–2011	Historical Cohort Study	PR(LRP)	0/0–1 pads	standard self-assessed questionnaire	N/A	99/99	1 mo: 33%/16% 3 mo: 66%/44% 6 mo: 81%/67%
Keiichi Ito^18^	Japan	2008–2011	Historical Cohort Study	PR(LRP)	0 pad	UCLA-PCI	mostly −	19/13	1 mo: 21%/7% 3 mo: 48%/13%
Chang Wook Jeong^19^	Korea	2009–2011	Historical Cohort Study	PR(RALP)	Complete: 0 pad Social: 0–1 pads	EPIC	mostly +	113/116	Complete: 2 wk: 30.1%/19.8% 1 mo: 58.4%/45.7% 3 mo: 82.7%/70.5%
Isaac Yi Kim^20^	USA	2007	Historical Cohort Study	PR(RALP)	0 pad	EPIC	N/A	25/25	1 wk: 24%/36% 3 mon: 84%/76%
Mike Nguyen^21^	USA	2006	Historical Cohort Study	PR(RALP/LRP)	0–1 pads	self-reported questionnaire	+	32/30	3 day: 34%/3% 6 wk: 56%/17%
Francesco Rocco^22^	Italy	1998–2005	Historical Cohort Study	PR(RRP)	0–1 pads	ICIQ-SF	+	250/50	3 day: 62.4%/14.0% 1 mon: 74.0%/30.0% 3 mon: 85.2%/46.0%
Takeshi Sano^23^	Japan	2007–2008	Historical Cohort Study	PR(LRP)	0 pad	N/A	+	25/23	1 mon: 44%/0% 3 mon: 60%/30.4%
Youn Chul You^24^	Korea	2008–2010	Historical Cohort Study	PR(RALP)	0–1 pads	ICQ	mostly −	28/31	1 mon: 57.2%/35.5%
James Brien^25^	USA	2006–2009	Historical Cohort Study	PR(RALP)	N/A	RAND-UCLA	mostly +	31/58	N/A
Tatsuo Gondo^26^	Italy	2006–2011	Historical Cohort Study	PR(RALP)	0 pad	N/A	−	85/16	1 mon: 67.1%/18.8%
Jason Woo^27^	USA	2008	Historical Cohort Study	PR(RALP)	0/0–1 pads	N/A	mostly +	69/63	median time to achieve continence: 90/150 day
Bernardo Rocco^28^	Italy	2005	Historical Cohort Study	PR(LRP)	0 pad	ICIQ-SF	+	31/31	3 day: 74.2%/25.8% 1 mon: 83.8%/32.3%
Spencer Krane^29^	USA	2007	Historical Cohort Study	PR(RALP)	0–1 pads	direct questionning	mostly +	42/42	2 mon: 85%/86%
Neil Joshi^30^	The Netherlands	2007–2008	Prospective Parallel Study (not RCT)	PR(RALP)	0 pad	EORTC-QLQ-C30 and PR25	+	53/54	3 mo: 24%/31%
Chang Wook Jeong^31^	Korea	2012–2013	Randomized Study	PR(RALP)	Complete: 0 pad Social: 0–1 pads	EPIC	+	50/45	2 wk: Complete: 24.0%/8.9% Social: 58.0%/37.8%
Douglas Sutherland^32^	USA	2008	Randomized Study	PR(RALP)	0–1 pads	EPIC and IPSS	mostly +	46/41	3 mon: 63%/81%
Yoshiki Sugimura^12^	Japan	1994–2000	Historical Cohort Study	AS(RRP)	0 pad	N/A	mostly +	24/22	1 wk: 50%/5% 1 mon: 75%/27%
Yoshiyuki Kojima^33^	Japan	2011–2012	Historical Cohort Study	AS(RALP)	1-hour pad test	IPSS, ICIQ-SF and EPIC	mostly −	27/30	1-hour pad test: 4 wk: 4.5 g/15.5 g
Vipul Patel^34^	USA	N/A	Historical Cohort Study	AS(RALP)	0 pad	EPIC	mostly +	237/94	1 mon: 40%/33% 3 mon: 92.8%/83%
Michael Campenni^35^	USA	1997–1998	Historical Cohort Study	AS(RRP)	0/0–1 pads	valsalva leak-point pressure	N/A	25/25	6 mon: complete:32%/12% social:76%/59%
Masanori Noguchi^36^	Japan	2001–2002	Historical Cohort Study	AS(RRP)	0 pad	UCLA-PCI	N/A	33/12	1 wk: 67%/0% 1 mon: 82%/25% 3 mon: 91%/50%
Masanori Noguchi^37^	Japan	2005–2006	Randomized Study	AS(RRP)	0 pad	UCLA-PCI	+	30/30	1 mon: 53%/20% 3 mon: 73%/47% 6 mon: 100%/83%
Jens-Uwe Stolzenburg^38^	Greece	2008–2009	Randomized Study	AS(LRP)	0–1 pads	EPIC and ICQ	mostly +	45/45	2 day: 11.1%/11.1% 3 mon: 81.3%/76.5%
Ashutosh Tewari^14^	Austria	2005–2007	Historical Cohort Study	AR+PR(RALP)	0 pad	EPIC and IPSS	+	182/518	1 wk: 38.27%/13.15% 3 mon: 91.3%/50.23%
Akio Hoshi^11^	Japan	2008–2012	Historical Cohort Study	AR+PR(LRP)	0–1 pads	EPIC	−	81/47	3 mo: 45.7%/26.1% 6 mo: 71.4%/46.8% 12 mo: 84.6%/60.9%
Nikolaos Koliakos^10^	Belgium	2007–2008	Randomized Study	AR+PR(RALP)	0 pad	ICIQ-SF	+	23/24	7 wk: 65.2/33.3%
Mani Menon^9^	USA	2007	Randomized Study	AR+PR(RALP)	0/0–1 pads	pad weighing	N/A	59/57	1 wk: Complete: 20%/16% Social: 54%/51%
Jonathan Kalisvaart^39^	USA	2003–2008	Historical Cohort Study	AS+PR(RALP)	0–1 pads	EPIC	mostly +	50/50	3 mo: 90.9%/48.2%
Xavier Hurtes^40^	France	2009–2010	Randomized Study	AS+PR(RALP)	0/0–1 pads	UCLA-PCI	mostly +	39/33	1 mo: 26.5%/7.1% 3 mo: 45.2%/15.4%

RRP = retropubic radical prostatectomy, RARP = robot-assisted radical prostatectomy, LRP = laparoscopic radical prostatectomy, PR = posterior reconstruction, AR = anterior reconstruction, AS = anterior suspension, IPSS = international prostate symptoms scores, EPIC = expanded prostate cancer index composite, ICIQ-SF = The international consultation on incontinence questionnaire-short form, ICQ = The international continence society questionnaire, UCLA-PCI = The university of California los angeles prostate cancer index, EORTC-QLQ-C30 = The European organization for research and treat ment of cancer quality of life-core 30, PR25 = The prostate cancer module, N/A = not available, S/C = study group/control group, +=done, − = not done.

### Effect of surgical technique on complete urinary continence rate

**Figure 2:**
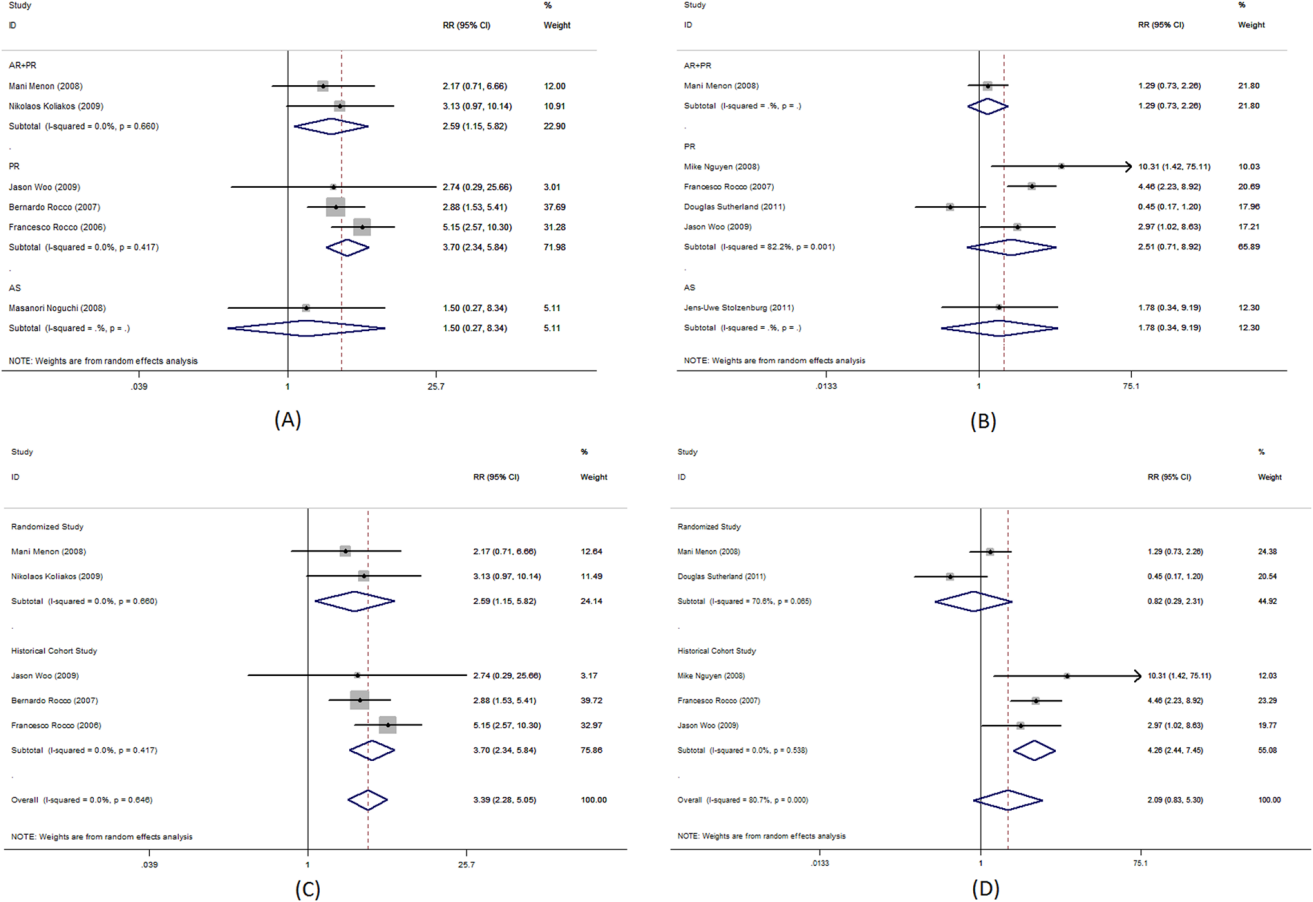
Forest plot of urinary continence across all studies at 1–4 days after catheter removal, (**A**) complete urinary continence; (**B**) social urinary continence; (**C**) complete urinary continence stratified by study design in studies including PR, AR + PR and AS + PR; (**D**) social urinary continence stratified by study design in studies including PR, AR + PR and AS + PR.

**Figure 3:**
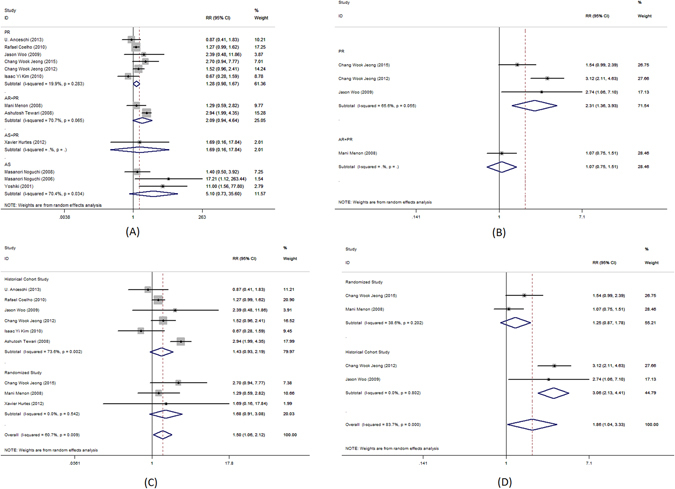
Forest plot of urinary continence across all studies at 28–42 days after catheter removal, (**A**) complete urinary continence; (**B**) social urinary continence; (**C**) complete urinary continence stratified by study design in studies including PR, AR + PR and AS + PR; (**D**) social urinary continence stratified by study design in studies including PR, AR + PR and AS + PR.

**Figure 4:**
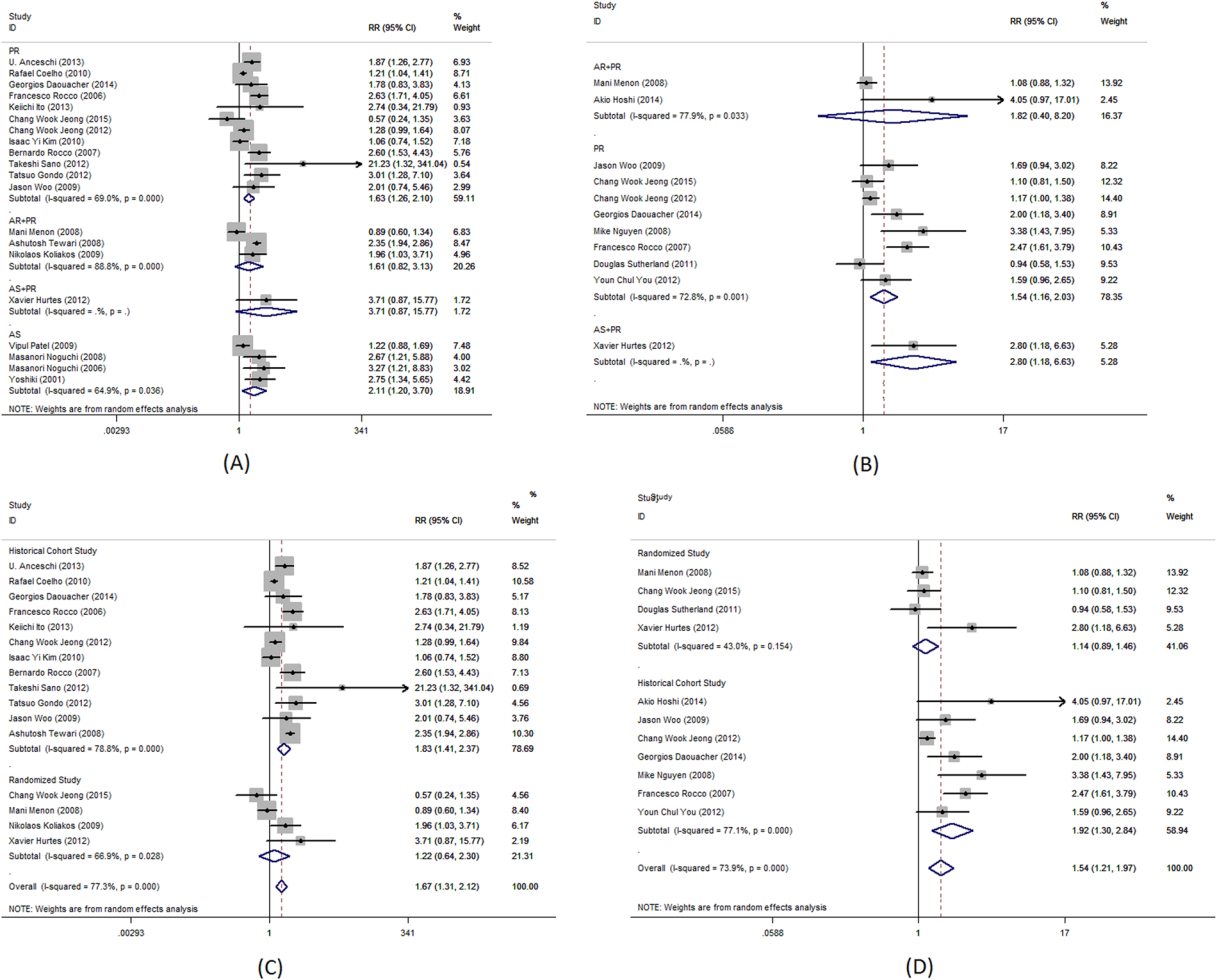
Forest plot of urinary continence across all studies at 90 days after catheter removal, (**A**) complete urinary continence; (**B**) social urinary continence; (**C**) complete urinary continence stratified by study design in studies including PR, AR + PR and AS + PR; (**D**) social urinary continence stratified by study design in studies including PR, AR + PR and AS + PR.

**Figure 5:**
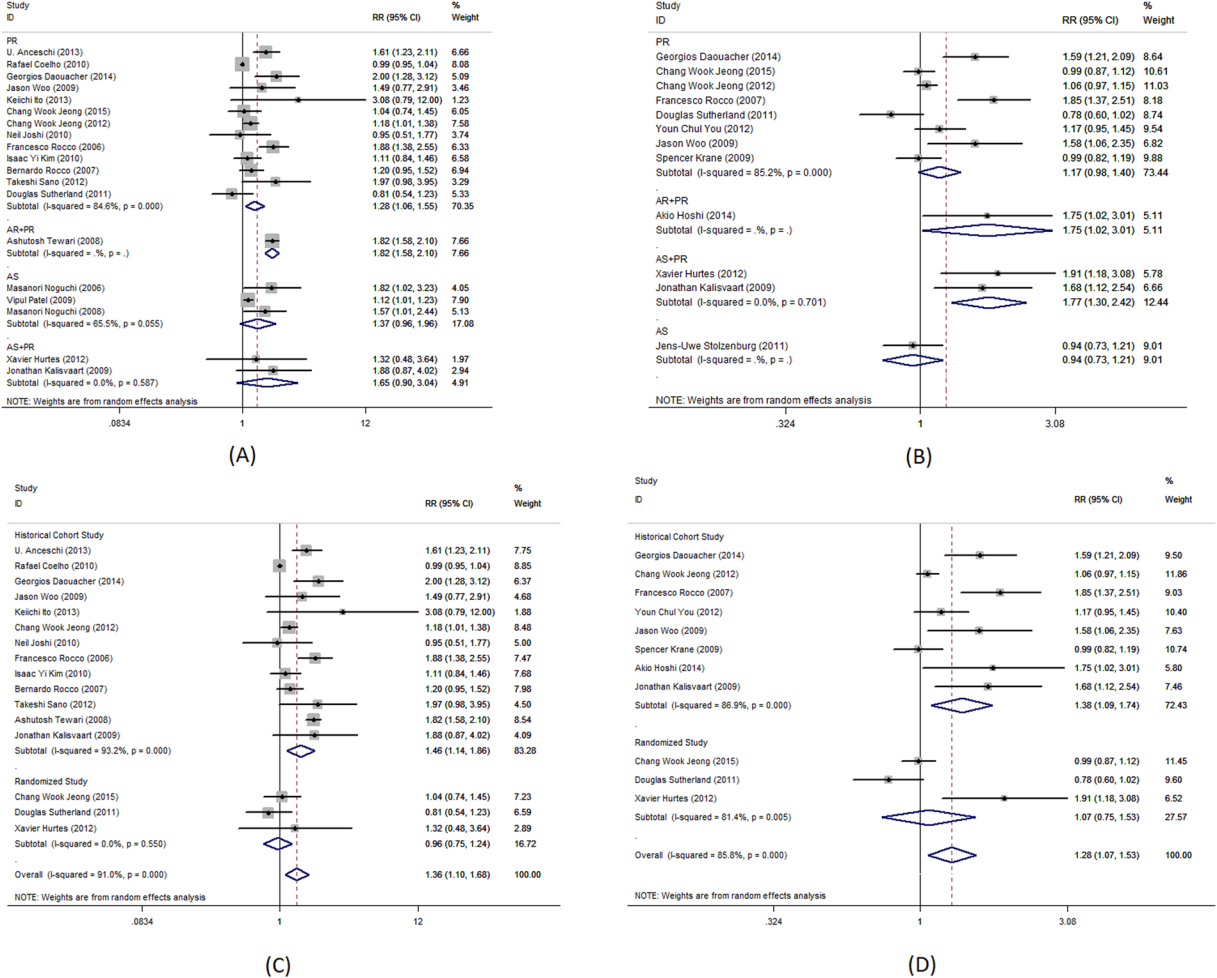
Forest plot of urinary continence across all studies at 180 days after catheter removal, (**A**) complete urinary continence; (**B**) social urinary continence; (**C**) complete urinary continence stratified by study design in studies including PR, AR + PR and AS + PR; (**D**) social urinary continence stratified by study design in studies including PR, AR + PR and AS + PR.

**Figure 6:**
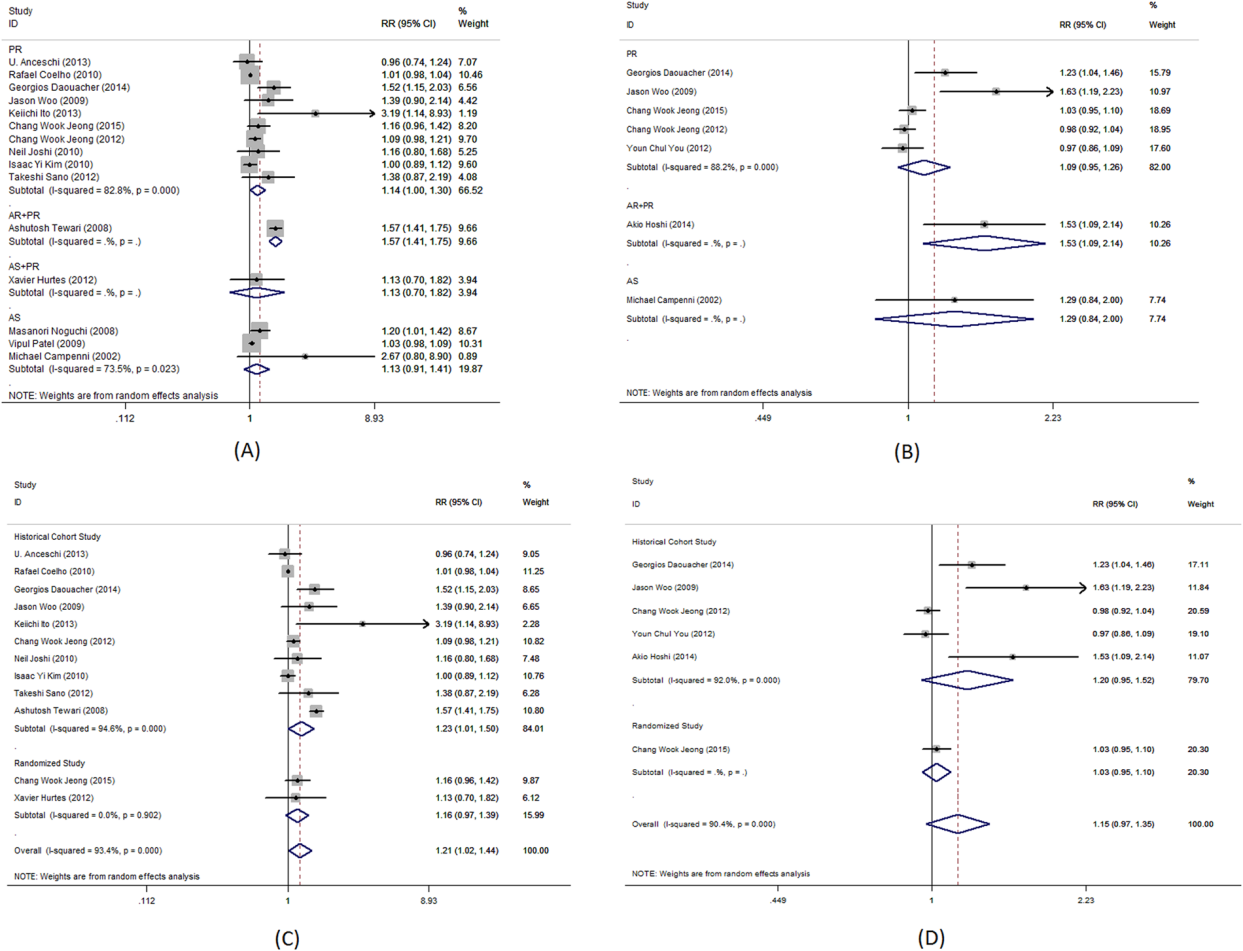
Forest plot of complete urinary continence across all studies at 360 days after catheter removal.

**Figure 7:**
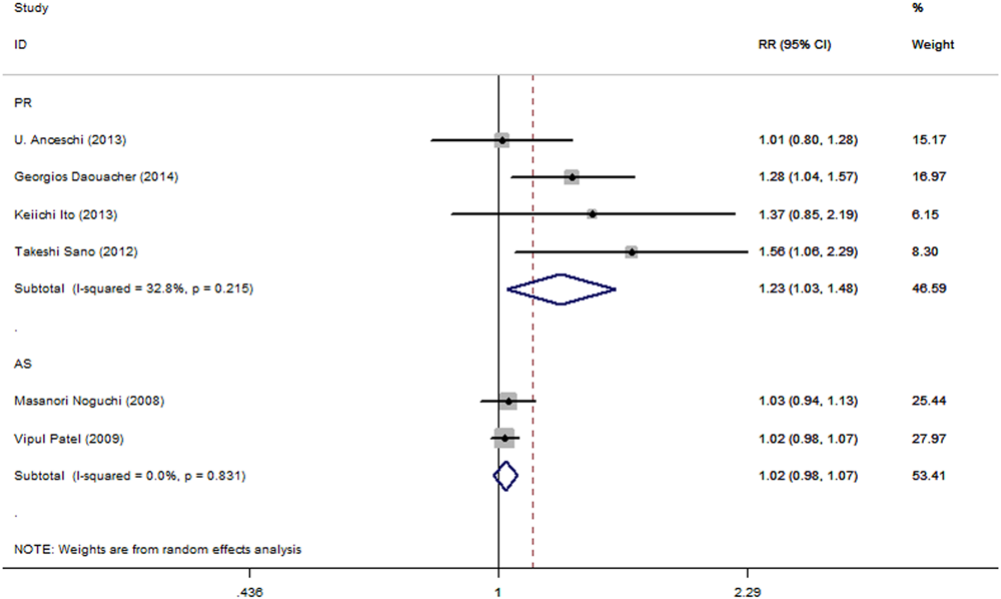
Forest plot of urinary continence across all studies at 7–14 days after catheter removal, (**A**) complete urinary continence; (**B**) social urinary continence; (**C**) complete urinary continence stratified by study design in studies including PR, AR + PR and AS + PR; (**D**) social urinary continence stratified by study design in studies including PR, AR + PR and AS + PR.

Complete urinary continence rate was the primary outcome measure in this meta-analysis. Pooled analysis of data showed that the use of PR alone was associated with significantly better complete urinary continence at 1–4, 28–42, 90, 180 and 360 days following the catheter removal (RR = 3.7; 95%CI, 2.34–5.84; P < 0.001, Fig. 2A; RR = 1.63; 95%CI, 1.26–2.1, P < 0.001, Fig. 3A; RR = 1.28; 95% CI, 1.06–1.55; P = 0.009, Fig. 4A; RR = 1.14; 95% CI, 1.00–1.30; P = 0.044, Fig. 5A; RR = 1.23; 95% CI, 1.03–1.48; P = 0.021, Fig. 6A, respectively). The use of PR was not associated with better complete urinary continence at 7 -14 days following catheter removal (RR = 1.28.; 95% CI, 0.98–1.67; P = 0.073, Fig. 7A).

The use of AS was associated with significantly better complete urinary continence at 28–42 days following the catheter removal (RR = 2.11; 95% CI, 1.20–3.70; P = 0.009, Fig. 4A). No benefit was identified 1–4, 7–14, 90, 180 or 360 days (RR = 1.5.; 95% CI, 0.27–8.34; P = 0.643, Fig. 2A; RR = 1.37; 95% CI, 0.96–1.96; P = 0.081, Fig. 4A; RR = 1.13; 95% CI, 0.91–1.41; P = 0.266, Fig. 5A; RR = 1.02; 95% CI, 0.98–1.07; P = 0.247, Fig. 6A; RR = 5.1.; 95% CI, 0.73–35.6; P = 0.100, Fig. 7A, respectively).

The use of AR + PR was associated with significantly better complete urinary continence at 1–4, 90 and 180 days following the catheter removal (RR = 2.59; 95% CI, 1.15–5.82; P = 0.022, Fig. 2A; RR = 1.82; 95% CI, 1.58–2.10; P < 0.001, Fig. 4A; RR = 1.14; 95% CI, 1.00–1.30; P < 0.001, Fig. 5A, respectively). However, no benefit was seen from AR + PR at 7–14 and 28–42 days following the catheter removal (RR = 1.61; 95% CI, 0.82–3.13; P = 0.163, Fig. 3A; RR = 2.09; 95% CI, 0.94–4.64; P = 0.069, Fig. 7A, respectively).

Complete urinary continence was similar in patients with and without AS + PR at 7–14, 28–42, 90 and 180 days (RR = 3.71; 95% CI, 0.87–15.77; P = 0.076, Fig. 3A; RR = 1.65; 95% CI, 0.90–3.04; P = 0.107, Fig. 4A; RR = 1.13; 95% CI, 0.70–1.82; P = 0.615, Fig. 5A; RR = 1.69; 95% CI, 0.16–17.84; P = 0.076, Fig. 7A, respectively).

The subgroup analysis of randomized trials evaluating PR, AR + PR and AS + PR demonstrated no improvement of complete urinary continence at 7–14, 28–42, 90 and 180 days after catheter removal (RR = 1.22; 95% CI, 0.64–2.30; P = 0.548, Fig. 3C; RR = 0.96; 95% CI, 0.75–1.24; P = 0.769, Fig. 4C; RR = 1.16; 95% CI, 0.97–1.39; P = 0.108, Fig. 5C; RR = 1.68; 95% CI, 0.91–3.08; P = 0.096, Fig. 7C, respectively). There was a significant improvement at 1–4 days after catheter removal (RR = 2.59; 95% CI, 1.15–5.82; P = 0.022, Fig. 1C). Historical cohort studies demonstrated a significant improvement of complete urinary continence at 1–4, 28–42, 90 and 180 days (RR = 3.70; 95% CI, 2.34–5.84; P < 0.001, Fig. 2C; RR = 1.83; 95% CI, 1.41–2.37; P < 0.001, Fig. 3C; RR = 1.46; 95% CI, 1.14–1.86; P = 0.003, Fig. 4C; RR = 1.23; 95% CI, 1.01–1.50; P = 0.041, Fig. 5C, respectively). No benefit was found at 7–14 days (RR = 1.43; 95% CI, 0.93–2.19; P = 0.104, Fig. 7C).

Reports where a nerve-sparing technique was not used had better complete urinary continence at 28–42 days (RR = 2.03; 95% CI, 1.35–3.06; P = 0.001, Figure S1), but no improvement 90 and 180 days(RR = 1.43; 95% CI, 0.96–2.14; P = 0.134, RR = 1.39; 95% CI, 0.85–2.77; P = 0.324, Figure S1, respectively).

### Effect of surgical technique on social urinary continence

Social urinary continence was a secondary outcome measure in this meta-analysis. Pooled analysis showed that the use of PR was associated with significantly improved social urinary continence at 7–14 and 28–42 days following catheter removal (RR = 1.54; 95% CI, 1.16–2.03; P = 0.003, Fig. 3B; RR = 2.31; 95% CI, 1.36–3.93; P = 0.002, Fig. 7B, respectively). No benefit was found at 1–4, 90 and 180 days (RR = 2.51; 95% CI, 0.71–8.92; P = 0.154, Fig. 2B; RR = 1.17; 95% CI, 0.98–1.40; P = 0.080, Fig. 4B; RR = 1.09; 95% CI, 0.95–1.26; P = 0.221, Fig. 5B, respectively).

Social urinary continence was not improved after AS at all time interval (1–4 days: RR = 1.78; 95% CI, 0.34–9.19; P = 0.493, Fig. 2B; 90 day: RR = 0.94; 95% CI, 0.73–1.21; P = 0.634, Fig. 4B; 180 day: RR = 1.29; 95% CI, 0.84–2.00; P = 0.247, Fig. 5B, respectively).

A significantly better outcome was observed after AR + PR at 90 and 180 days after catheter removal (RR = 1.75; 95% CI, 1.02–3.01; P = 0.043, Fig. 4B; RR = 1.53; 95% CI, 1.09–2.14; P = 0.014, Fig. 5B, respectively). No benefit was found at 1–4, 7–14 and 28–42 days (RR = 1.29; 95% CI, 0.73–2.26; P = 0.377, Fig. 2B; RR = 1.82; 95% CI, 0.40–8.20; P = 0.436, Fig. 3B; RR = 1.07; 95% CI, 0.75–1.51; P = 0.717, Fig. 7B, respectively).

Data was available evaluating the use of AS + PR at 28–42 and 90 days after catheter removal. The use of AS + PR significantly improved social urinary continence (28–42 days: RR = 2.80; 95% CI, 1.18–6.63; P = 0.019, Fig. 3B; 90 days: RR = 1.77; 95% CI, 1.30–2.42; P < 0.001, Fig. 4B, respectively).

Analysis of randomized trials evaluating PR, AR + PR and AS + PR demonstrated no improvement of social urinary continence at 1–4, 7–14, 28–42, 90 and 180 days after catheter removal (RR = 0.82; 95% CI, 0.29–2.31; P = 0.708, Fig. 2D; RR = 1.14; 95% CI, 0.89–1.46; P = 0.314, Fig. 3D; RR = 1.07; 95% CI, 0.75–1.53; P = 0.715, Fig. 4D; RR = 1.03; 95% CI, 0.95–1.10; P = 0.506, Fig. 5D; RR = 1.25; 95% CI, 0.87–1.78; P = 0.226, Fig. 7D, respectively). Historical cohort studies showed a significant benefit in social urinary continence at 1–4, 7–14, 28–42 and 90 days (RR = 4.26; 95% CI, 2.44–7.45; P < 0.001, Fig. 2D; RR = 1.92; 95% CI, 1.30–2.84; P = 0.001, Fig. 3D; RR = 1.38; 95% CI, 1.09–1.74; P = 0.007, Fig. 4D; RR = 3.06; 95% CI, 2.13–4.41; P < 0.001, Fig. 7D, respectively). No benefit was seen at 180 days (RR = 1.20; 95% CI, 0.95–1.52; P = 0.131, Fig. 5D).

### Effect of surgical treatment on PSM and cystogram leakage

**Figure 8:**
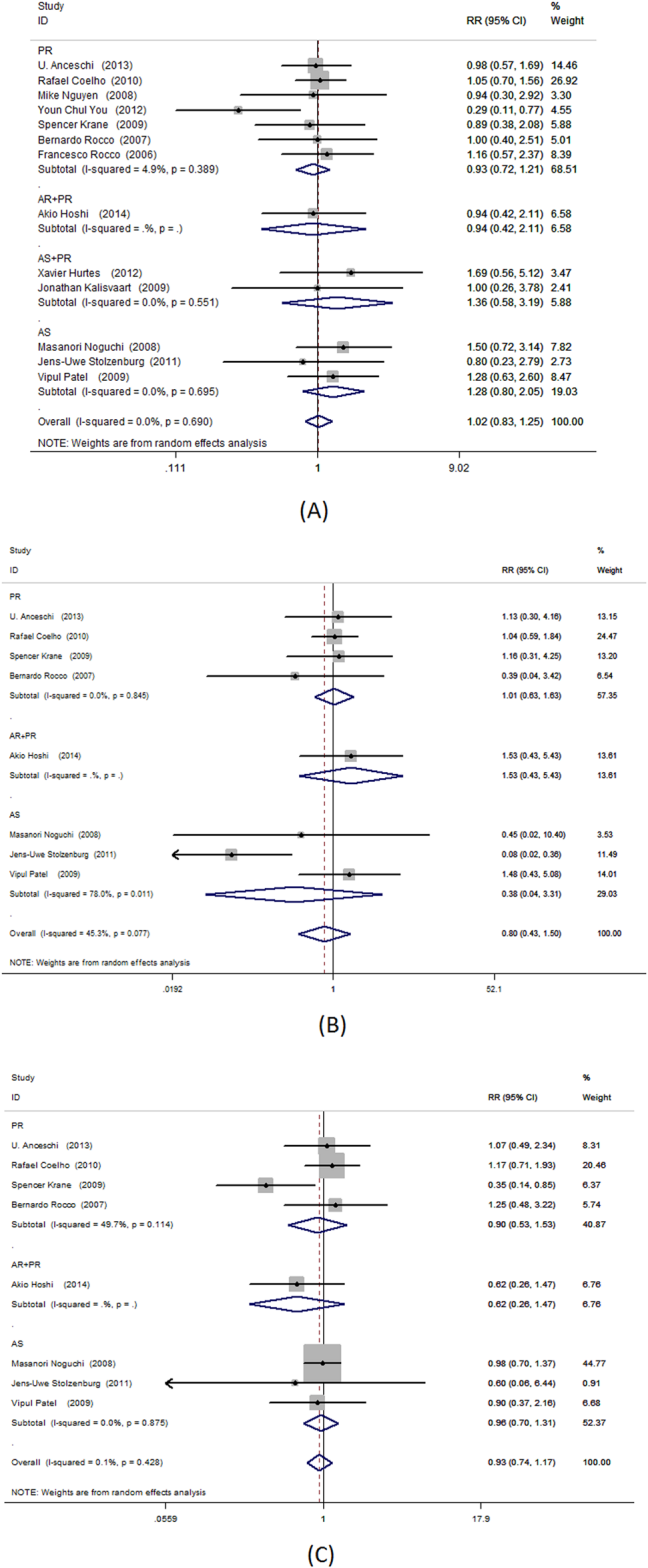
Forest plot of PSM rate, (**A**) all patients (**B**) patients with pT2; (**C**) patients with pT3.

Thirteen trials evaluated PSM rate, including seven for PR, three for AS, one for AR + PR and two for AS + PR. No differences were observed in the PSM rates associated with each surgical technique (PR: RR = 0.93; 95% CI, 0.72–1.21; P = 0.604; AS: RR = 1.28; 95% CI, 0.80–2.05; P = 0.312; AR + PR: RR = 0.94; 95% CI, 0.42–2.11; P = 0.886; AS + PR: RR = 1.36; 95% CI, 0.58–3.19; P = 0.474, Fig. 8A, respectively).

PSM rates did not vary by surgical technique in patients with stage pT2 cancer (PR: RR = 1.01; 95% CI, 0.63–1.63; P = 0.951; AS: RR = 0.38; 95% CI, 0.04–3.31; P = 0.382; AR + PR: RR = 1.53; 95% CI, 0.43–5.43; P = 0.511, Fig. 8B, respectively). PSM rates also did not vary by surgical technique in patients with stage pT3 cancer (PR: RR = 0.90; 95% CI, 0.53–1.53; P = 0.693; AS: RR = 0.96; 95% CI, 0.70–1.31; P = 0.802; AR + PR: RR = 0.62; 95% CI, 0.26–1.47; P = 0.275, Fig. 8C, respectively).

**Figure 9:**
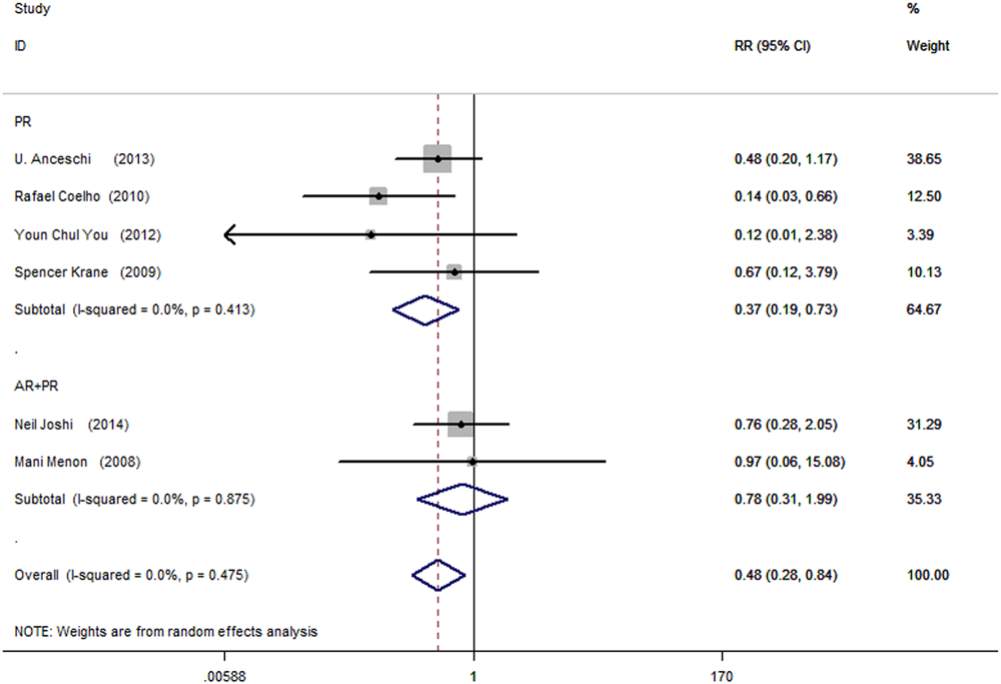
Forest plot of urinary leakage at postoperative cystogram.

Pooled data from 6 trials showed PR was associated with the least amount of cystogram leakage after surgery (RR = 0.37; 95% CI, 0.19–0.73; P = 0.004, Fig. 9). No significant benefit was detected in patients after AR + PR (RR = 0.78; 95% CI, 0.31–1.99; P = 0.610, Fig. 9).

### Effect of surgical treatment on IPSS and EPIC urinary domain scores

IPSS and EPIC urinary domain scores were reported in six studies^11, 18, 25, 29, 32, 33^. Kojima *et al*.^33^ reported a median IPSS score before surgery of 12.5 in the AS group and 7.0 in the control group. These values were 11.0 and 16.0, respectively, 4 weeks after surgery (P < 0.05). No benefit was also seen at week 12 or week 24. Sutherland *et al*.^32^ reported that both the PR and control groups had a significantly improved IPSS score from postoperative week 6 to month 3 (P < 0.01). Krane *et al*.^29^ found no difference in the IPSS score of the AS and control groups (8.2 vs 8.1, P = 0.97).

“Urinary function” and “urinary bother” subscale score from the EPIC urinary domain were also reviewed. Hoshi *et al*.^11^ found that the proportion of recovery to baseline score was significantly improved in the “urinary function” subscale score at 12 months after surgery (P < 0.01) No significant improvement was found at other time points for the “urinary function” or at any time point for the “urinary bother” subscale score. Different outcomes were reported by Ito *et al*.^18^ and Brien *et al*.^25^. Both found “urinary function” and “urinary bother” subscale scores to be significantly higher in the PR treated group, compared to a control group, at 3 months after surgery. Ito *et al*.^18^ found a significant improvement in “urinary function” and “urinary bother” subscale scores at 6 months after surgery when PR was performed. In contrast, Brien *et al*. reported no benefit in these scores 6 months after catheter removal^25^.

### Quality assessment of RCTs and historical cohort studies

The Jadad quality scores and methodological Newcastle-Ottawa scales are listed in Table 2. The quality of cohort studies was mostly high, but the level of evidence was low because of the nature of the study designs. Because of the lack of double blind for a surgery, the score for double blind in mostly studies was 0, expect one^9^. The quality of most RCTs was still high, and the level of evidence was stable expect one study^38^.

**Table 2:** The methodological Newcastle-Ottawa scales, Jadad quality scores and level of evidence assessment of the included observational studies.

Historical cohort study (Newcastle-Ottawa Scale)					
Author(Year)	Selection	Comparability	Outcome	Total score	Level of evidence
U. Anceschi(2013)	***	*	**	6	4
Rafael Coelho(2010)	****	**	**	8	2b
Georgios Daouacher(2014)	****	**	**	8	2b
Keiichi Ito(2013)	***	*	**	6	4
Chang Wook Jeong(2012)	****	**	**	8	4
Neil Joshi(2010)	****	**	**	8	2b
Isaac Yi Kim(2010)	***	**	**	7	4
Mike Nguyen(2008)	***	**	**	7	4
Francesco Rocco(2007)	***	*	**	6	4
Takeshi Sano(2012)	***	*	**	6	2b
Youn Chul You(2012)	***	**	**	7	4
James Brien(2011)	****	**	**	8	3b
Tatsuo Gondo(2012)	****	**	**	8	4
Jason Woo(2009)	****	**	**	8	2b
Spencer Krane(2009)	***	*	**	6	4
Bernardo Rocco(2007)	***	**	**	7	2b
Francesco Rocco(2006)	***	**	**	7	4
Yoshiyuki Kojima(2014)	***	**	**	7	4
Vipul Patel(2009)	****	**	**	8	4
Michael Campenni(2002)	***	*	**	6	4
Masanori Noguchi(2006)	***	*	**	6	4
Yoshiki Sugimura(2001)	***	*	**	6	4
Akio Hoshi(2014)	****	**	**	8	4
Ashutosh Tewari(2008)	****	*	**	7	4
Jonathan Kalisvaart(2009)	***	**	**	7	4
**Randomized controlled trial (Jadad score)**					
**Author(Year)**	**Randomized**	**Double blind**	**Withdrawals and dropouts**	**Total score**	**Level of evidence**
Chang Wook Jeong(2015)	2	0	1	3	1b
Douglas Sutherland(2011)	2	0	0	2	1b
Masanori Noguchi(2008)	2	0	1	3	1b
Jens-Uwe Stolzenburg(2011)	1	0	0	1	2b
Mani Menon(2008)	2	2	1	5	1b
Nikolaos Koliakos(2009)	2	0	1	3	1b
Xavier Hurtes(2012)	2	0	1	3	1b

### Publication bias

Funnel plots of urinary continence at six time intervals showed only one publication with bias, in the AS treated group at 28–42 days (Begger test P = 0.089, Egger test P = 0.002). This bias could be due to the small number of patients with follow-up. No evidence of publication bias was found at any time interval with the other surgical treatments used (Figs S2–S8) (Table 3).

**Table 3:** Pooled results of complete urinary continence, social urinary continence, PSM rates and publication bias of comparing different surgical techniques and time points.

Outcome measures	n	No. Patient R/NR	Pooled RR (95% CI)	Hterogeneity		Begg’s test(P)	Egger’s test(P)
				I^2^(%)	P		
**Complete urinary continence**							
**PR modification**							
1–4 day	3	261/144	3.7(2.34–5.84)	0.0	0.417	0.296	0.194
7–14 day	6	781/633	1.28(0.98–1.67)	19.9	0.283	1.000	0.963
28–42 day	12	1201/865	1.63(1.26–2.1)	69.0	<0.001	0.350	0.185
90 day	13	1215/944	1.28(1.06–1.55)	84.6	<0.001	0.428	0.372
180 day	10	977/822	1.14(1.00–1.30)	82.8	<0.001	1.000	0.612
360 day	4	195/189	1.23(1.03–1.48)	32.8	0.215	0.734	0.499
**AS modifcation**							
7–14 day	3	87/64	5.1(0.73–35.6)	70.4	0.034	1.000	N/A
28–42 day	3	324/158	2.11(1.20–3.70)	64.9	0.036	0.089	**0**.**002**
90 day	3	300/136	1.37(0.96–1.96)	65.5	0.055	0.296	0.227
180 day	3	292/149	1.13(0.91–1.41)	73.5	0.023	1.000	N/A
**AR** + **PR modification**							
28–42 day	3	264/599	1.61(0.82–3.13)	88.8	<0.001	1.000	0.642
**Social urinary continence**							
**PR modification**							
1–4 day	4	397/184	2.51(0.71–8.92)	82.2	0.001	1.000	0.872
7–14 day	3	232/224	2.31(1.36–3.93)	65.6	0.055	1.000	0.453
28–42 day	8	687/475	1.54(1.16–2.03)	72.8	0.001	1.000	0.931
90 day	8	692/487	1.17(0.98–1.40)	85.2	<0.001	0.266	0.169
180 day	5	359/354	1.09(0.95–1.26)	88.2	<0.001	0.462	0.361
**PSM rate**							
PR modification	7	819/568	0.93(0.72–1.21)	4.9	0.389	0.133	0.299
AS modifcation	3	312/169	1.28(0.80–2.05)	0.0	0.695	1.000	0.725

## Discussion

This meta-analysis included 7 randomized studies and 25 historical cohort studies of different urethral reconstruction methods after radical prostatectomy, including PR, AS, PR + AS and PR + AR. A quantitative synthesis of the evidence can be really helpful for urologist because urinary incontinence is the major problem after radical prostatectomy.

Urinary incontinence could be improved by many techniques, such as pelvic floor reconstruction, bladder neck preservation^41^ or intussusceptions^42^, preserving the fascia covering the levator ani muscle^43^ and preserving neurovascular bundles^44^. Among these techniques, pelvic floor reconstruction was reported most. The reconstruction prolonged a little surgery time and gained benefit in improving urinary continence. And the hot point for reconstruction is which layers to be sutured and how to suture. So many studies used different methods to improve the urinary continence compared to the common technique in this meta-analysis.

Patients were evaluated at a large number of time points for both complete and social continence, and a large number of surgical techniques were evaluated. Evaluation of pooled results demonstrated an improvement in urinary continence using these techniques. PR group outcomes in this meta-analysis were similar to the results in Rocco *et al*.^13^, but two different points should be noticed. First, we analyzed complete continence and social continence, respectively. Second, we used 1–4, 7–14, 28–42, 90, 180 and 360 day after catheter removal as cut-off point. Meanwhile, no differences in PSM and cystogram leakage were identified.

Treatment of patients with PR improved the complete urinary continence rate at 0–4, 28–42, 90, 180 and 360 days after catheter removal, but not at 7–14 days. These findings are similar to those reported by Grasso *et al*.^8^ and Rocco *et al*.^13^. Rocco *et al*.^13^ found no improvement in the urinary continence rate at 3 and 6 months after catheter removal. This finding was similar to the improvement in social urinary continence rate seen with the pooled data. The different inclusion criteria used and different number of trials evaluating different outcomes could have contributed to some of the different findings. AS provided no benefit of complete or social urinary continence, except at 28–42 days after catheter removal. AR + PR and AS + PR did not show significant benefit until 180 or more days after catheter removal.

There are some kinds of potential heterogeneity in this meta-analysis. First, surgical technical differences were reported in each of the surgical reconstructions, although these were felt to be minor. For example, Patel *et al*.^34^ anchored the anastomosis to the pubic bone, while Noguchi *et al*.^36^ anchored to the dorsal venous complex (DVC) and puboprostatic ligaments. Second, different methods were used to evaluate continence including a self-administrated questionnaire, EPIC questionnaire, valsalva leak-point pressure, and pad weighing. Third, different study designs including the variable use of a nerve-sparing technique, variations in reporting times, and differences in the historical cohorts used as control groups could have influenced the outcomes. We did not distinguish randomized studies from historical cohort studies because of the small number of reported trials. Finally, the difference in the number of patients treated in each study could introduce bias into our analysis. These potential effects make high heterogeneity of results. It’s impossible to control these differences in each trial.

Bias due to different study designs may be greater in subgroup analyses. Both complete and social urinary continence was present only at 1–4 days in RCTs, where heterogeneity was generally low. Complete urinary incontinence was observed at 7–14 days and social urinary incontinence at 180 days in historical studies. These differences could occur because RCTs better control patient related bias and also because there may be small differences in the surgical technique used in the two groups. The IPSS and EPIC urinary domain score was analyzed in this meta-analysis. Because the scale scores were not well described using RR, and so were individually described by report. This is another method to assess the postoperative urinary continence.

There were several limitations to this study. First, only publications reported in English were included because of the lack of a translator. Second, the individual patient data was not available for each study which is the gold standard for meta-analysis. Third, conference abstracts were also not included because of lack of available data. These factors could have reduced the number of trials evaluated in this meta-analysis. Fourth, heterogeneity and variation in study quality, as described above, could also have affected results. Lastly, different time intervals among the included studies also influenced the outcomes despite of grouping sections. These limitations may make the results unstable, so further studies are still needed to explore the effect of these surgical techniques in RP.

## Conclusion

Patients with PCa who underwent RP with PR had the least urinary incontinence. PR is currently one of the most widely used surgical reconstructive techniques to improve the adverse effect of RP. No benefit was observed after AS. AR + PR, while AS + PR, might have little influence at early time points, but had the best outcomes at 180 or more days. More RCTs are needed to better assess the efficacy of different surgical reconstructions after RP.

## Methods

### Selection Criteria

Studies that were published in English were selected if they met the following criteria: (1) all patients were diagnosed with PCa by clinical examinations and prostate biopsy; (2) all patients underwent radical prostatectomy; and (3) the surgical modification was AS, AR, PR, AS + PR or AR + PR. Studies of patients who received neoadjuvant treatment were excluded.

### Search Strategy

This meta-analysis was conducted following the Preferred Reporting Items for Systematic Reviews and Meta-Analyses (PRISMA) statement^15^. To identify studies that met the above selection criteria, we searched the PubMed, Embase, Cochrane Central Register of Controlled trials, Ovid and Web of Science databases for trials published before June 6, 2016. The search strategy was followed using all possible combinations of the medical subject headings (MeSH) or non-MeSH terms including prostate neoplasm, prostatic neoplasm, and prostatic cancer; posterior reconstruction, anterior reconstruction, anterior suspension, pelvic floor reconstruction and total reconstruction; urinary incontinence and incontinence or urinary continence and continence. Each search strategy was conducted in each database. We also manually searched for potentially relevant trials from the references of studies identified by the above search.

### Data extraction

Two reviewers (JF Cui and Hu Guo) independently assessed all eligible publications. Any discrepancies were settled by discussion with a third reviewer (BK Shi). Data that met the selection criteria were collected on a standardized form by two independent reviewers. Data extracted from the studies included the author’s name, publication year, country, study period, study design, surgical technique, definition of continence, method for evaluation of continence, use of nerve sparing techniques, number of patients and results, including risk ratios [RRs], 95% confidence intervals [CIs] and P values.

### Outcome Measures

The primary outcome measure in this meta-analysis was complete urinary continence rate. Complete urinary continence was defined as using 0 pad per day. The secondary outcome measure was social urinary continence. Social urinary continence was defined as using 0–1 pads per day. The study group was defined as the group with one kind of reconstruction which not mentioned in the control group. The control group was defined as the group without the reconstruction which mentioned in study group. Continence rates were determined at 1–4, 7–14, 28–42, 90, 180 and 360 days after catheter removal. Positive surgical margin (PSM) rate, leakage on cystogram, international prostate symptoms scores (IPSS) and expanded prostate cancer index composite (EPIC) urinary domain score were also determined.

### Statistical Analysis

RRs with 95% CIs were used to evaluate the primary outcome and secondary outcome. A RR > 1 indicated an advantage of reconstruction over non-reconstruction (NR). Heterogeneity across studies was quantified using the I^2^ statistic and the Chi-square (Cochrane Q statistic) test. Studies with an I^2^ statistic greater than 40% and a P value less than 0.1 for the Chi-square test had a high level of heterogeneity. A random-effects model was used to pool estimates regardless of high or low levels of heterogeneity in order to better deal with the heterogeneous nature of the different surgical modifications. Study designs, surgical modifications and other confounding factors were not consistent between studies. Therefore, there was a significant advantage of a random-effects model compared with a fixed-effects model in accounting for heterogeneity between studies^16^. A p value less than 0.05 was considered statistically significant. All statistical analyses were performed using STATA version 13.0 (College Station, Texas, USA).

### Quality Assessment

The methodological quality of each randomized controlled trial (RCT) was evaluated using the Jadad scale^17^. Quality was assessed using presence of randomization (0–2 points), used of double blind (0–2 points) and presence of patient withdrawals and dropouts (0–1 point). The 2 reviewers classified studies into two quality grades: low (0–2 points) and high (3–5 points).

The methodological quality of each cohort study was evaluated according to the Newcastle-Ottawa Scale (NOS)^18^. Method of selection of the study groups (0–4 points), comparability of cohorts (0–2 points) and ascertainment of the outcome (0–3 points) were the three major aspects used for calculating the quality score of included reports. The studies were classified into three quality grades: low (0–3 points), moderate (4–6 points) or high (7–9 points). All studies were evaluated using the level of evidence (LOE) defined by Phillips *et al*.^19, 45–49^. Two independent reviewers evaluated each study. Disagreements were resolved through discussion.

## Electronic supplementary material

Supplementary material
