# Supplementary material for: Pelvic Floor Reconstruction After Radical Prostatectomy: A Systematic Review and Meta-analysis of Different Surgical Techniques
Source: Sci Rep. 2017 Jun 2;7:2737. doi: 10.1038/s41598-017-02991-8 (PMC5457408; doi:10.1038/s41598-017-02991-8)
Supplement: Supplementary file 1 — Supplementary material [file 41598_2017_2991_MOESM1_ESM.pdf]

Title Page

Pelvic Floor Reconstruction After Radical Prostatectomy: A Systematic Review and Meta-analysis of Different Surgical Techniques

Jianfeng Cui[1], Hu Guo[1], Yan Li[1], Shouzhen Chen[1], Yaofeng Zhu[1], Shiyu Wang[1], Yong Wang[1], Xigao Liu[1], Wenbo Wang[2], Jie Han[3], Pengxiang Chen[4], Shuping Nie[5], Gang Yin[1*], Benkang Shi[1*]

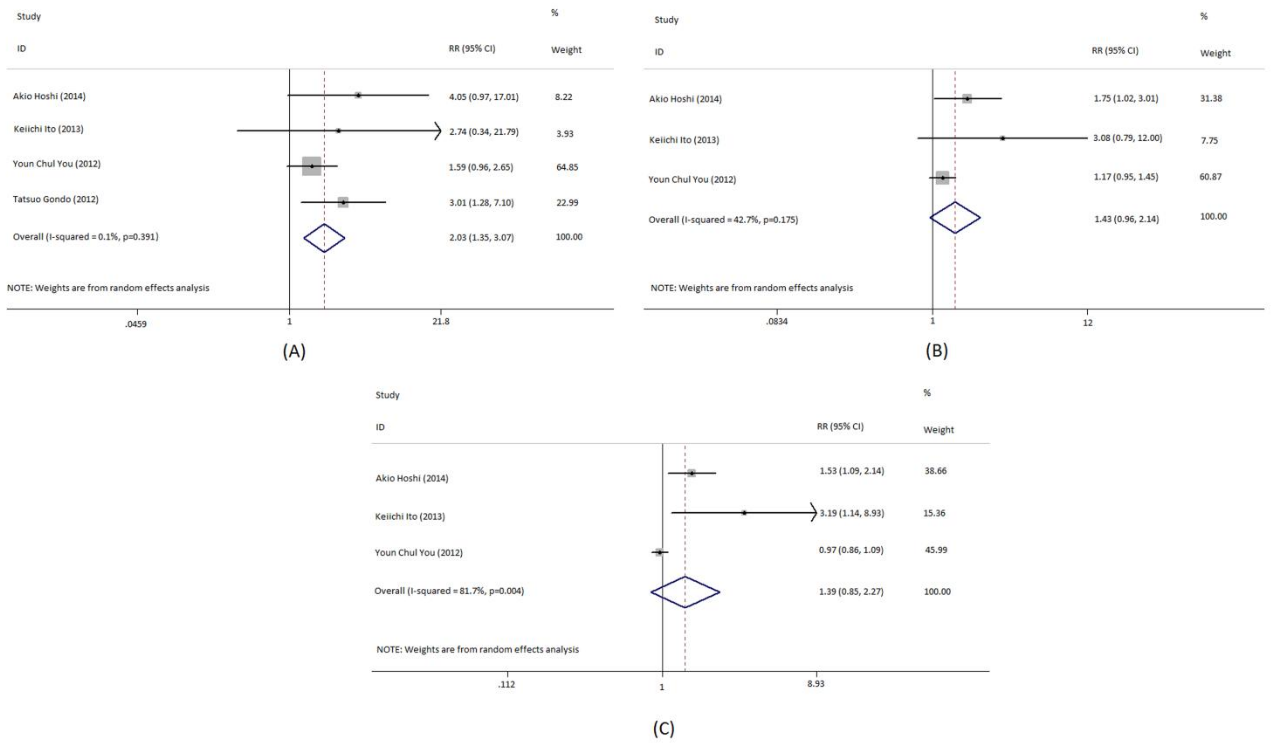

Fig.S1 Forest plot of urinary continence across studies which didn't spare the nerve, (A) 28-42 days after catheter removal; (B) 90 days; (C) 180 days after catheter removal

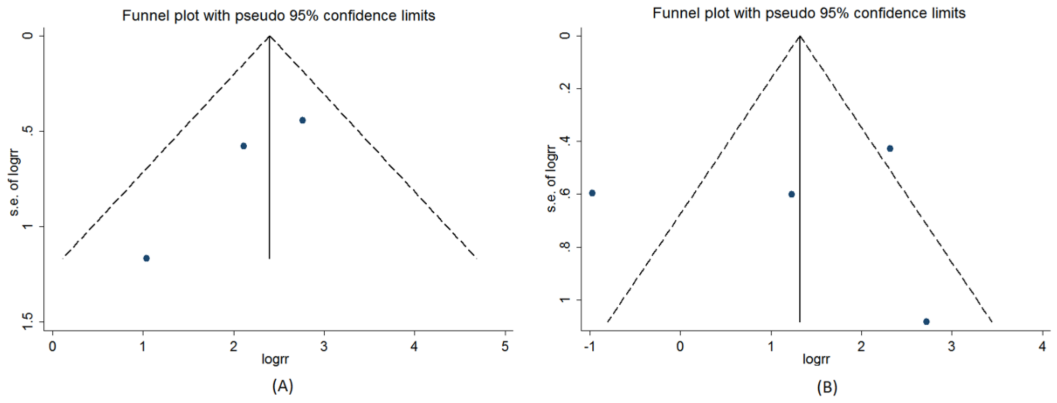

Fig.S2 Funnel plot assessing publication bias across all studies at 1-4 days after catheter removal, (A) complete urinary continence for PR; (B) social urinary continence for PR

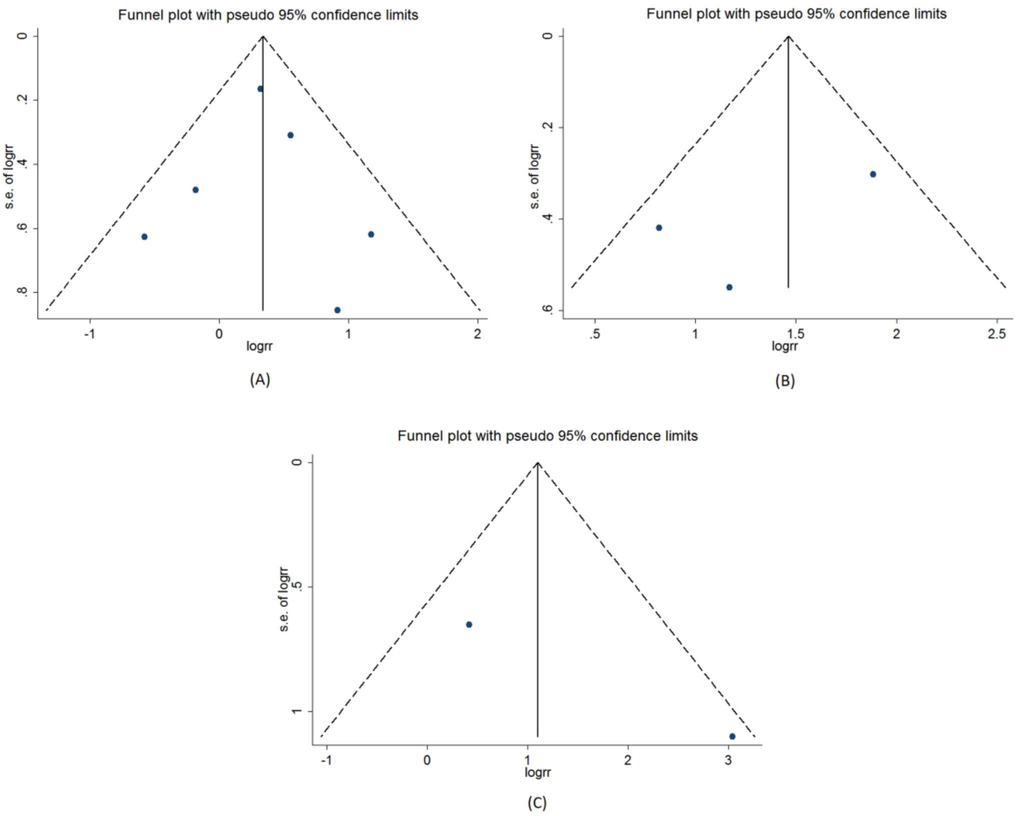

Fig.S3 Funnel plot assessing publication bias across all studies at 7-14 days after catheter removal, (A) complete urinary continence for PR; (B) social urinary continence for PR; (C) complete urinary continence for AS

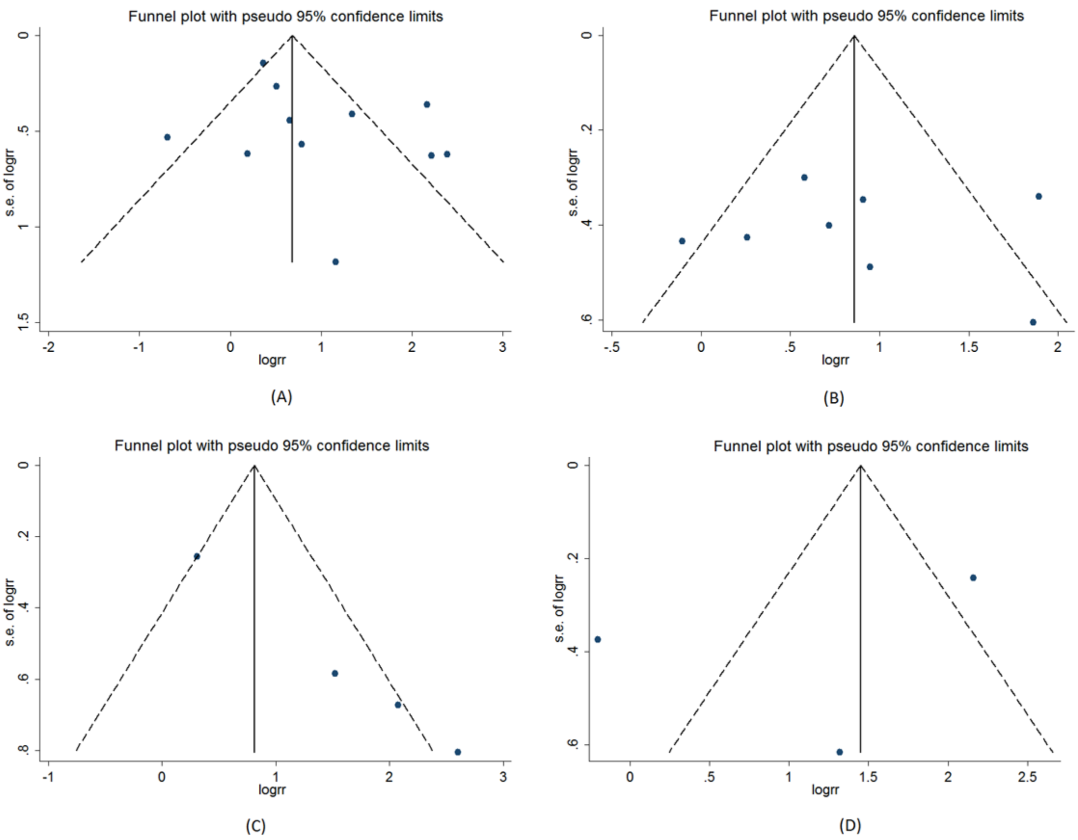

Fig.S4 Funnel plot assessing publication bias across all studies at 28-42 days after catheter removal, (A) complete urinary continence for PR; (B) social urinary continence for PR; (C) complete urinary continence for AS; (D) complete urinary continence for AR+PR

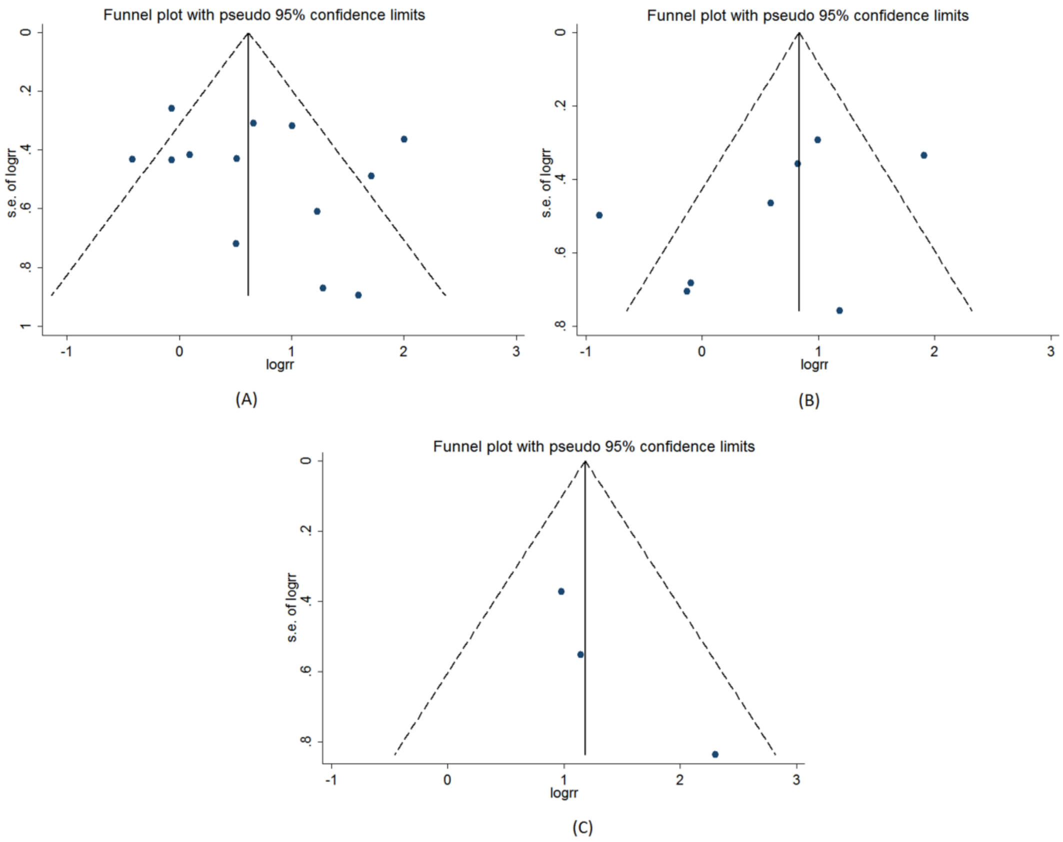

Fig.S5 Funnel plot assessing publication bias across all studies at 90 days after catheter removal, (A) complete urinary continence for PR; (B) social urinary continence for PR; (C) complete urinary continence for AS

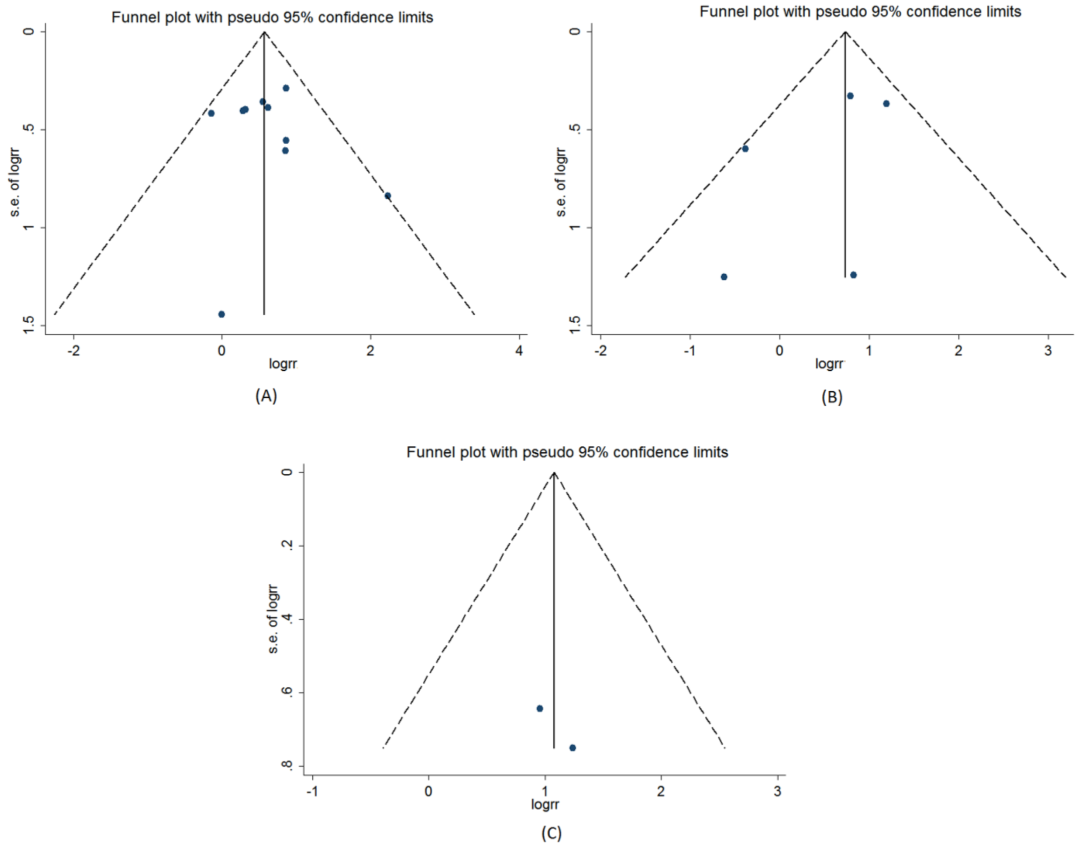

Fig.S6 Funnel plot assessing publication bias across all studies at 180 days after catheter removal, (A) complete urinary continence for PR; (B) social urinary continence for PR; (C) complete urinary continence for AS

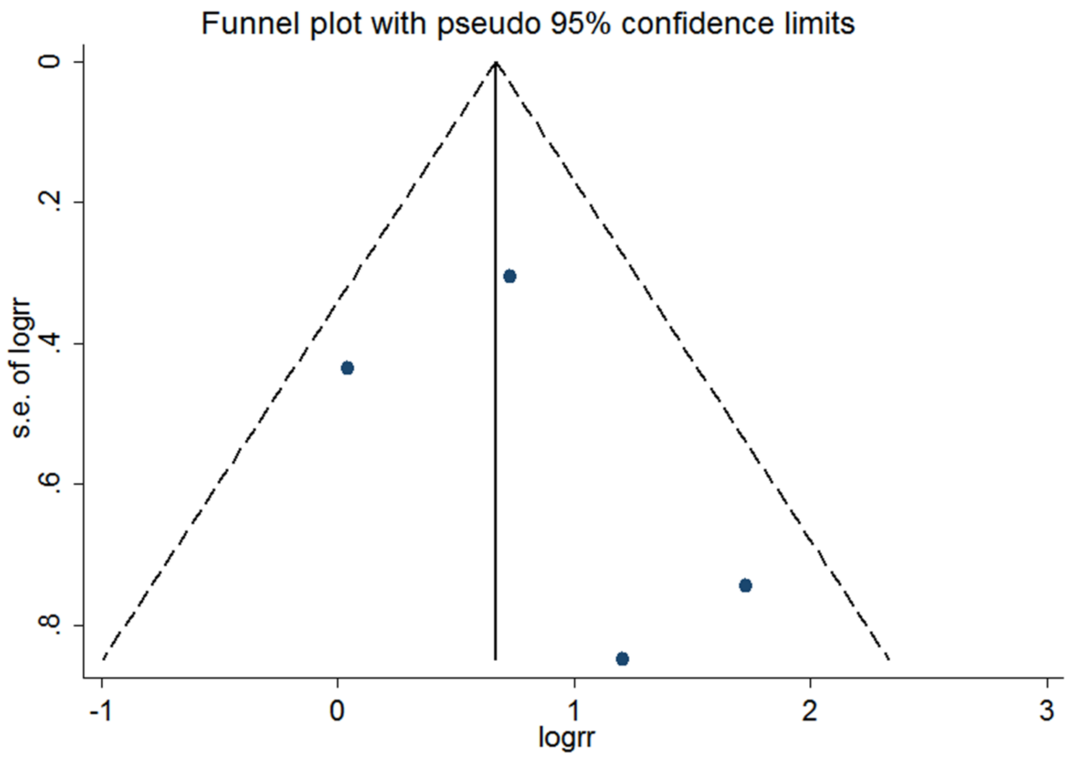

Fig.S7 Funnel plot assessing publication bias of complete urinary continence for PR across all studies at 360 days after catheter removal

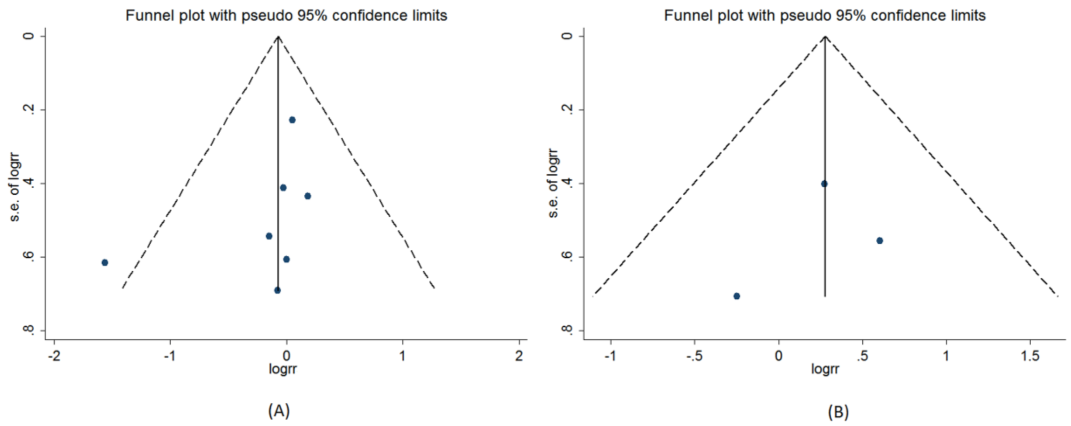

Fig.S8 Funnel plot assessing publication bias across all studies for PSM rate, (A) PR; (B) AS
